# Prospective Analysis Comparing the Asthma Control Test (ACT) to the New Subjective Asthma Questionnaire (SAQ-1) for Assessing Asthma Control

**DOI:** 10.7759/cureus.72357

**Published:** 2024-10-25

**Authors:** Bryan K Dunn, Kori L Brewer, Keven O'Brien, Veeranna Maddipati, Messanh K Ameduite, Anagha Malur

**Affiliations:** 1 Pulmonary and Critical Care, East Carolina University Brody School of Medicine, Greenville, USA; 2 Emergency Medicine, East Carolina University Brody School of Medicine, Greenville, USA; 3 Biostatistics, East Carolina University, Greenville , USA; 4 Pulmonary and Critical Care, East Carolina University Health Medical Center/East Carolina University Brody School of Medicine, Greenville, USA

**Keywords:** asthma, asthma control test (act), global initiative for asthma (gina) guidelines, national asthma education and prevention program (naepp) guidelines, subjective asthma questionnaire-1 (saq-1)

## Abstract

Background

Asthma affects millions of patients worldwide. The Global Initiative for Asthma (GINA) emphasizes the need for individualized treatment based on symptoms and risk of exacerbations. The Asthma Control Test (ACT) is a well-validated tool and considered standard-of-care for assessing asthma control. Our research question was to determine the correlation of the new Subjective Asthma Questionnaire-1 (SAQ-1) with the established ACT for assessing asthma control, and what are the associations of demographic and clinical variables with both scoring systems.

Study Design and Methods

In this prospective observational study, ACT, SAQ-1 scores, demographic and clinical characteristics were obtained during the pulmonary outpatient clinic visits of 115 adult patients with mild, moderate, and severe persistent asthma.

Results

Our research observed correlations between the ACT and SAQ-1 scores. Asthma severity was negatively associated with both ACT and SAQ-1 scores, while race had a weaker negative association with both scores. The SAQ-1 was found to have excellent sensitivity and specificity for assessing not well-controlled asthma.

Conclusion

The SAQ-1 is a valid and simple alternative to the ACT for assessing asthma control and may improve patient compliance and enable more frequent assessments.

## Introduction

Asthma is caused by airway hyperresponsiveness and inflammation which is triggered by reversible airway bronchoconstriction from an immune response to allergens, and affects 25 million patients in the United States (US) [1,2]. In 2021, for adults and children in the US, the overall prevalence of asthma exacerbations was 98,000 cases per year, and 3,517 patients died from asthma [1]. Making having a simple and effective scoring system to assess asthma control crucial for patient care.

The 2023 Global Initiative for Asthma (GINA) and National Asthma Education and Prevention Program (NAEPP) guidelines recommend that asthma treatment should be individualized based on symptom frequency, symptom severity, and overall risk of exacerbations [2,3]. The primary goals of asthma management are symptom relief, prevention of acute asthmatic attacks, and improvement of quality of life [2,3]. Multiple scoring systems are used to assess asthma control, including the Asthma Control Test (ACT), Asthma Control Questionnaire (ACQ)-5, 6, and 7, and the recently released Asthma Impairment and Risk Questionnaire (AIRQ) [4-6].

The most commonly used tool in the US is the standard-of-care ACT score, which is well-validated for assessing asthma control and is used to determine treatment strategies based on 2023 GINA guidelines [2,6]. The total ACT score ranges from 5 to 25 and assesses control over the past 4 weeks [2,6,7]. The self-administered ACT consists of five questions scored using a 5-point Likert scale (1-5), comprising two on asthma symptoms, one on the use of rescue inhalers, one on interference with daily activities, and one on self-perception of asthma control [6,8,9].

Schatz et al. conducted a longitudinal study to assess the reliability and validity of the ACT compared to the ACQ among new patients of asthma specialists in outpatient settings in the United States [10]. They stated that patients tend to overestimate their asthma control [10]. They found that an ACT score <19 had the best sensitivity (71%) and specificity (71%) and was useful for determining asthma control (without the need for pulmonary function testing) [10]. Schatz et al. also reported ACT score ranges and categorized asthma control levels [9-10]. An ACT score was 25, 20-24, 16-19, and <15 and was completely controlled, well-controlled, not well-controlled, and poorly controlled asthma, respectively [9-10]. Zhou et al. evaluated the accuracy of the ACT score in primary care settings in China [11]. They reported an internal consistency reliability of 0.861 and a correlation coefficient of 0.697 when comparing ACT scores with asthma severity, which did show a moderate-to-strong relationship. Zhou et al. established the ACT score to categorize asthma control levels [11]. An ACT score includes≥20, 18-19, and ≤17 which correlated with well-controlled, partially controlled, and uncontrolled asthma, respectively. These ACT score thresholds were adopted in clinical practice to help with treatment decisions and patient outcomes [11].

Regarding the creation of the ACT, Nathan et al. published “Development of the asthma control test: a survey for assessing asthma control” in 2004 in The Journal of Allergy and Clinical Immunology, which discusses its development and validation [8]. Their goal was to create a straightforward method for assessing asthma control without the need for pulmonary function testing. The study reviewed the results of a 22-item survey (including spirometry) of 471 asthmatic patients. As mentioned earlier, they selected five key questions to form the ACT [8]. Schatz et al. found that the minimally important difference in the ACT score was 3 points [9]. The ACT score system and other asthma scoring systems may be unfamiliar and difficult to use for non-pulmonary physicians and other providers managing asthma patients and using an easier scoring system, such as our new Subjective Asthma Questionnaire-1 (SAQ-1), may be more practical.

Nathan et al. stated that patients may overestimate the control of their asthma symptoms [8]. However, during their development of the ACT score, they found that patients could evaluate their own asthma control, based on item number 6 of the 22 analyzed, which corresponds to ACT item number 5 (“How would you rate your asthma control during the past 4 weeks”, which is scored on a 1-5 Likert scale) [8,12]. This was based on logistic regression (the dependent variable was a binary variable reflecting a specialist’s rating of asthma control; “not controlled” was assigned a value of 1), which gave a significant odds ratio of 0.68 (p = 0.0002).

In this prospective study, we explored the utility of our new, single-item SAQ-1 to the ACT score. The SAQ-1 score is similar to the ACT score item number 5 as is a self-reported item question. SAQ-1 score grades on a scale from 0-10 rate how well-controlled your asthma has been over the last 4 weeks; 0 indicates very poorly controlled asthma and 10 indicates very well-controlled asthma. Both the SAQ-1 and ACT evaluate asthma control over a 4-week period, but the ACT involves five questions, while the SAQ-1 only involves the patient’s perception of their asthma control. We propose that the SAQ-1 may be used alone or alongside the current standard-of-care ACT scoring system [6-8, 10]. The SAQ-1 may be easier to use in the asthma scoring system due to its simple one-question format. The SAQ-1 may improve patient compliance by reducing the time and effort required to answer the questionnaire. Also, the SAQ-1 may help with more frequent assessments by providers and patients who are not familiar with the current asthma questionnaires, such as the ACT score.

The research questions were as follows: What is the correlation of the new, simple, single-item SAQ-1 score with the established standard-of-care multi-item ACT score for assessing asthma control, and what are the associations of demographic and clinical variables with both scoring systems? Our main goal was to validate the efficacy of using the SAQ-1 to assess asthma control as part of standard-of-care practices in outpatient settings, which may allow for more frequent assessments. The patient population under study comprised patients attending an academic pulmonary outpatient clinic.

The research hypothesis was as follows: The SAQ-1 score strongly correlates with the ACT score and can effectively assess asthma control with similar sensitivity and specificity to the ACT, making the SAQ-1 score an alternative for routine clinical use.

## Materials and methods

This study was conducted in accordance with the amended Declaration of Helsinki. The protocol for this cross-sectional, prospective, single-center study comparing the ACT score with the SAQ-1 score was reviewed by the University Medical Center and Institutional Review Board and certified as exempt under category #4C (UMCIRB 24-000333).

Study subjects and data collection

We prospectively collected data on 122 patients with a diagnosis of mild, moderate, or severe persistent asthma who presented at our academic pulmonary outpatient clinic over a 1-year period (April 2023 to April 2024). There were 115 patients included in the final analysis.

The inclusion criteria comprised adult patients aged greater than 18 with a diagnosis of mild, moderate, or severe persistent asthma. The exclusion criteria comprised no formal diagnosis of asthma, age >80, not actively receiving treatment for asthma, diagnosis of interstitial lung disease (ILD), chronic pulmonary infections (viral, fungal or bacterial, *Mycobacterium avium* complex or *Mycobacterium tuberculosis*), requirement for oxygen to maintain oxygen saturations >90%, and incomplete ACT and SAQ-1 scores or medical records.

**Figure 1:**
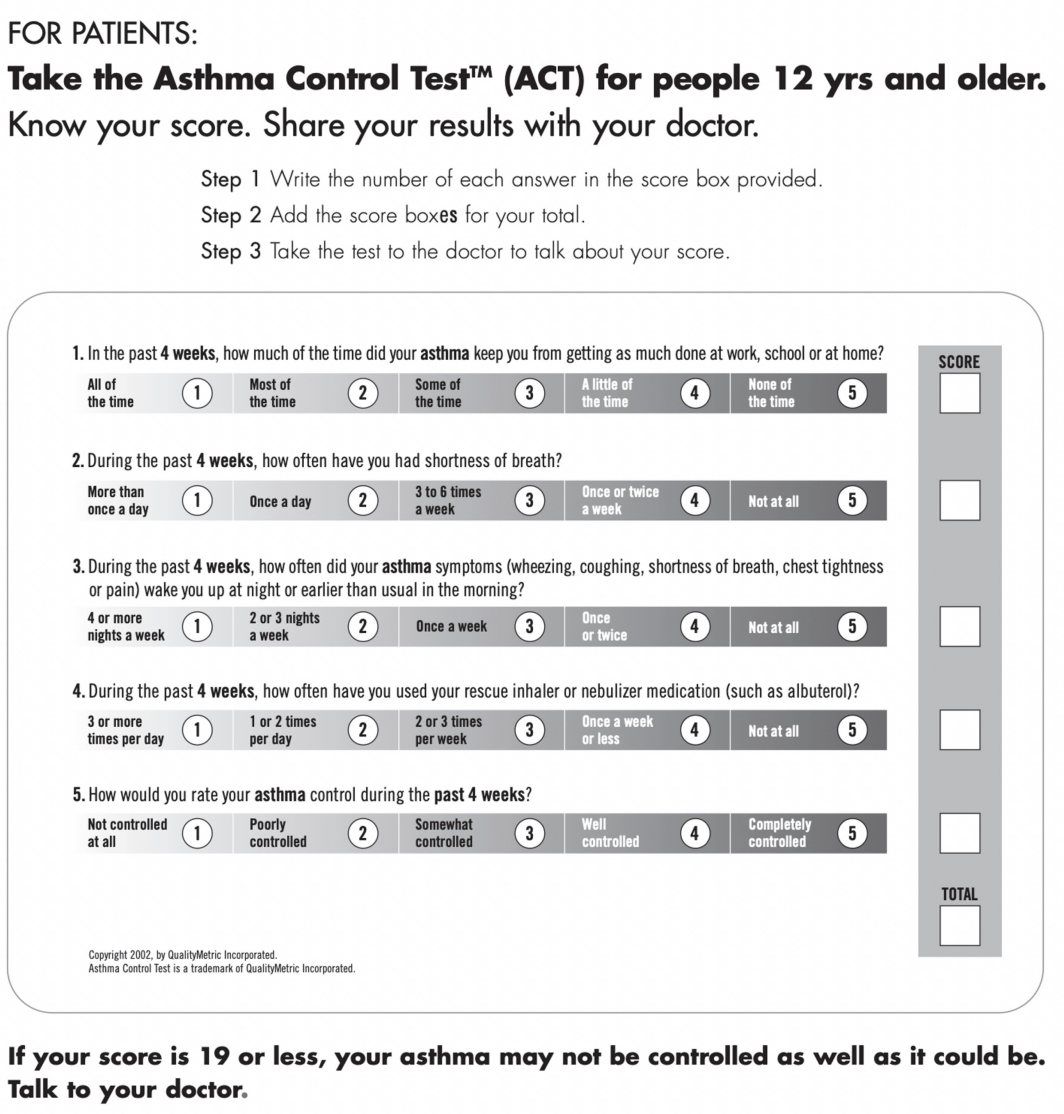
The Asthma Control Test™ (ACT) ranging from 5 to 25. A trademark of Quality Metric Incorporated. The ACT is provided by GlaxoSmithKline (GSK) and was developed by Nathan et al. and is available (open access) online. The use of the ACT is compliant with copyright guidelines.

**Figure 2:**
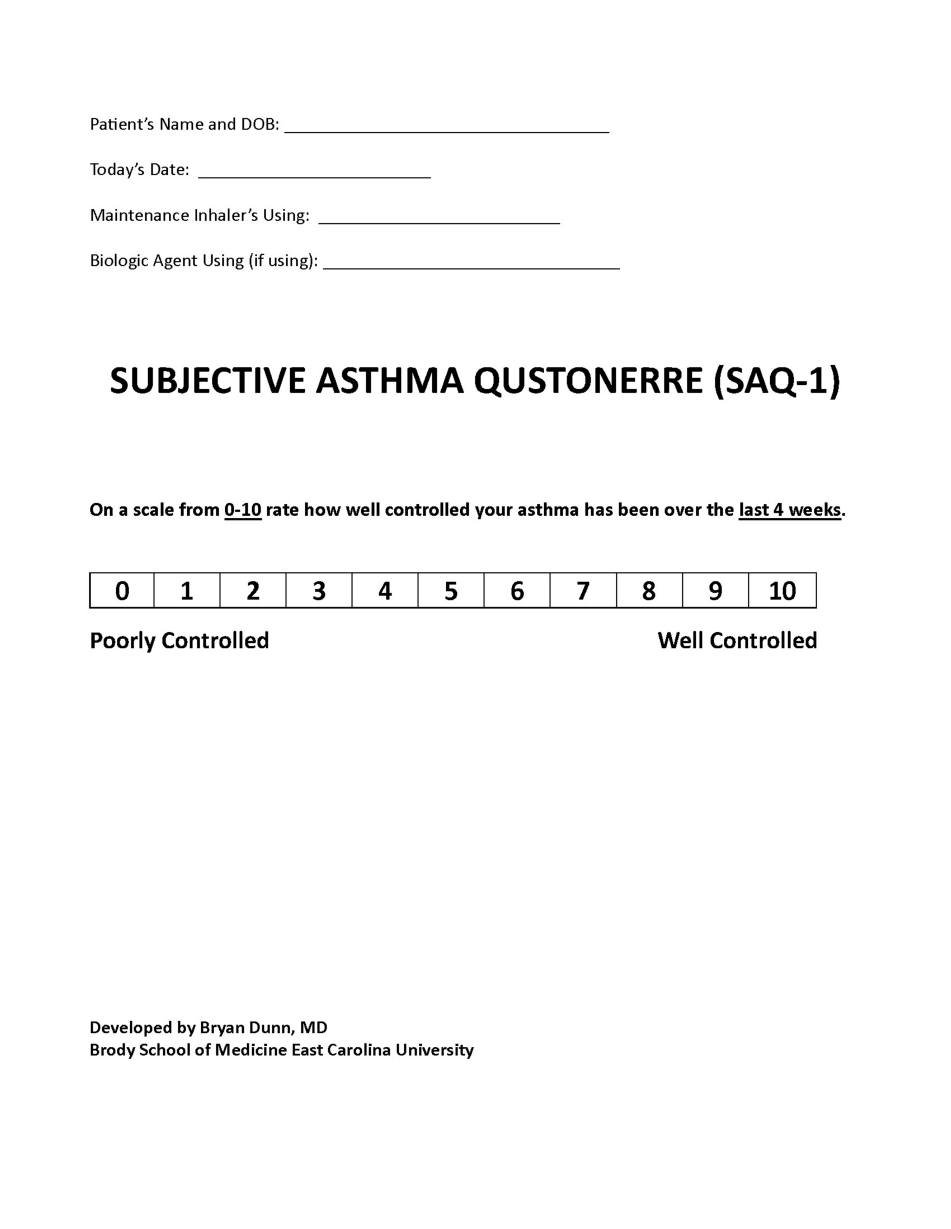
Subjective Asthma Questionnaire-1 (SAQ-1), ranging from 0-10. Developed by East Carolina University Brody School of Medicine, Bryan K. Dunn, MD.

Data were collected by a faculty pulmonary physician who specializes in treating asthma patients. The data comprised ACT and SAQ-1 scores and demographic and clinical characteristics. The demographic characteristics were gender, age, and race. The clinical characteristics were weight, body mass index (BMI), smoking status, and pulmonary function tests, i.e., forced expiratory volume in 1 second (FEV1), forced vital capacity (FVC), total lung capacity (TLC), residual volume (RV), and diffusion capacity of the lungs for carbon monoxide (DLCO), asthma severity, and elevated serum eosinophils. The ACT and SAQ-1 scores were determined during each patient’s pulmonary outpatient clinic visit using paper surveys self-administered by the patient (Figures 1-2). The demographic and clinical characteristics were obtained from electronic medical records (Epic Systems, Verona, WI, USA).

Primary and secondary outcomes

The primary outcome was the correlation between the SAQ-1 and ACT scores. The secondary outcomes were the associations of the demographic variables (gender, age, and race) and clinical variables (weight, BMI, smoking status, pulmonary function tests, asthma severity, and elevated serum eosinophils) with the ACT and SAQ-1 scores.

Statistical analysis

The statistical analysis was conducted using IBM SPSS Statistics for Windows v29.0.2.0 (IBM Corp., Armonk, NY, USA). First, descriptive statistics were calculated. Second, Pearson, Kendall’s Tau-b, and Spearman correlation analyses were used to assess the correlation between ACT and SAQ-1 scores; this approach was chosen to accommodate the rank-based properties of the data. Third, a cross-tabulation analysis was used to further explore the relationship between ACT and SAQ-1 scores. Fourth, a receiver operating characteristic (ROC) curve analysis was used to assess the discriminatory ability of SAQ-1 scores. Fifth, a scatter plot was used to visualize the relationship between ACT and SAQ-1 scores by gender. Lastly, Pearson correlation was used to assess whether categorical demographic and clinical variables were associated with ACT or SAQ-1 scores.

Pearson’s correlation was chosen because it measures the degree to which one variable projects onto another, making it suitable for assessing the relationship between continuous variables. Although Pearson’s correlation assumes normality for valid p-values, the central limit theorem ensures that with a large enough sample, the sampling distribution approaches normality, validating the p-values. In our analysis, QQ plots were used to assess normality, and the data were deemed sufficiently close to justify the use of Pearson’s correlation. Additionally, the consistency of results across Pearson, Spearman, and Kendall correlations supports the appropriateness of using Pearson’s correlation, given its widespread understanding and interpretability.

## Results

Study sample

The final study population (Tables 1-2) consisted of 115 patients, 30 (25.9%) were male and 85 (74.1%) were female, showing a predominantly female population of asthma patients seen in our clinic. The mean age was 52, with a range of 18 to 78 years. For asthma severity, 18 (15.5%) of patients had mild persistent asthma, 27 (23.3%) had moderate persistent asthma, and 70 (61.2%) had severe persistent asthma. For race, 86 (75.0%) of the patients identified as Black and 29 (25.0%) as White, which showed the demographics of the patient population seen at our pulmonary clinic. For smoking history, 66 (57.7%) were non-smokers and 44 (38.6%) were prior smokers and 4 (3.4%) currently smoke. For elevated serum eosinophils, 67 (58.6%) of the patients had elevated serum eosinophils, a common marker associated with eosinophilic asthma.

**Table 1:** Continuous demographic and clinical variables (n = 115 patients). FEV1: forced expiratory volume in 1 second; FVC: forced vital capacity; TLC: forced vital capacity; RV: residual volume; DLCO: diffusion capacity of the lungs for carbon monoxide

Variable	Minimum	Maximum	Mean	Median	95% CI
Age (years)	18	78	52	50	49.4–54.7
Weight (kg)	43	207	103	100	97.0–108.8
BMI (kg/m^2^)	18	70	37	35	34.7–38.4
FEV1/FVC (%)	42	102	74	72	71.0–76.7
FEV1 (liters)	0.86	3.60	2	1.9	1.8–2.1
FEV1 (%)	32	126	73.6	72	69.3–77.8
FVC (liters)	1.21	4.7	2.6	2.5	2.3–2.7
FVC (%)	44	126	81	80	77.7–84.5
TLC (%)	61	164	95	93	90.2–98.9
RV (%)	51	199	117	115	109.7–124.2
DLCO corrected (%)	21	184	82.9	80	77–88.9

**Table 2:** Categorical demographic and clinical variables (n = 115 patients).

Variable	Categories	Percentages (%)
Asthma classification	Mild/Moderate/Severe persistent	18 (15.5%)/27 (23.3%)/70 (61.2%)
Elevated serum eosinophils	Yes/No	67 (58.6%)/48 (41.4%)
Gender	Male patients/Female patients	30 (25.9%)/85 (74.1%)
Race	Black patients/White patients	86 (75.0%)/29 (25.0%)
Smoking status	Never/Former/Active	66 (57.7%)/44 (38.6%)/4 (3.4%)

Primary analysis

**Figure 3:**
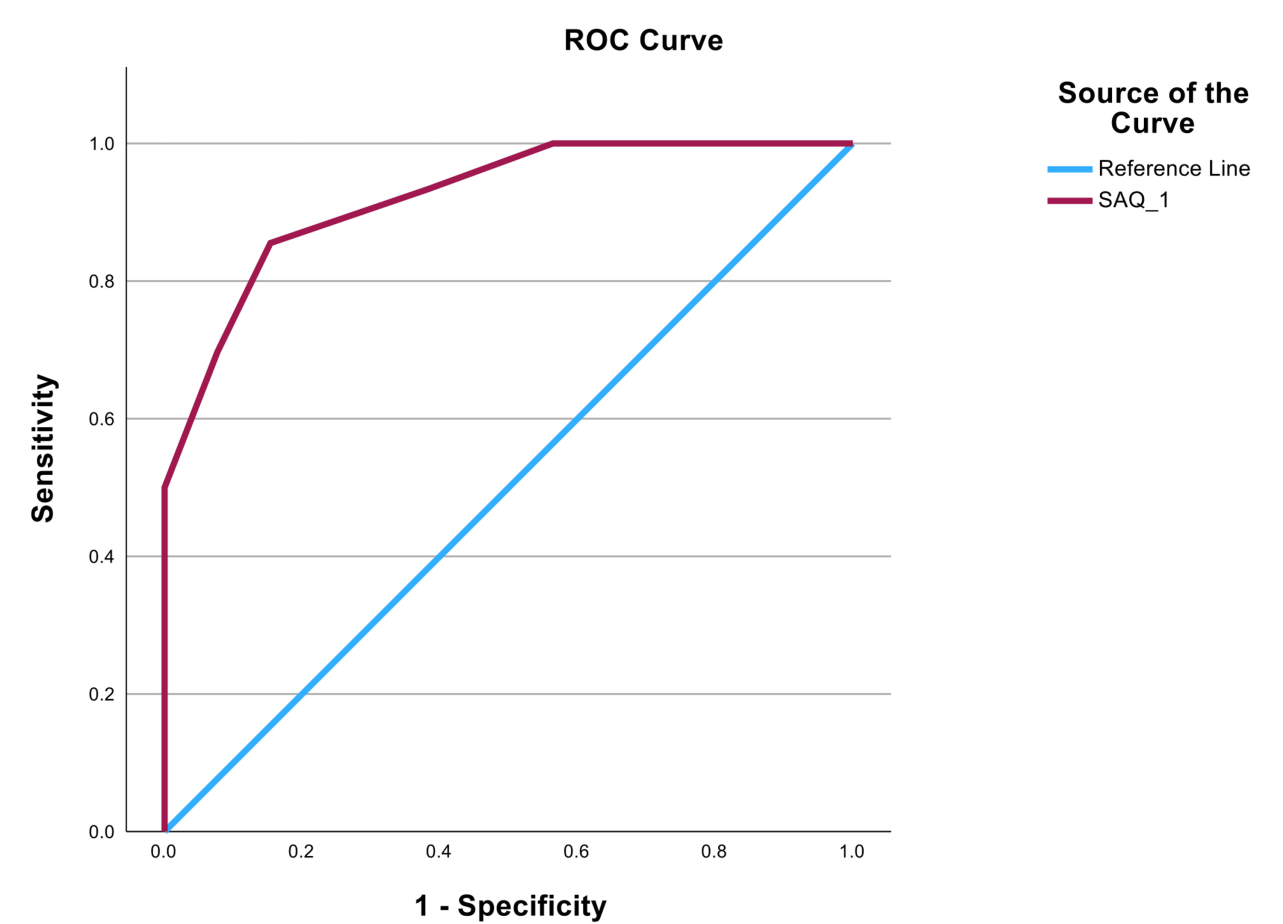
ROC curve analysis. The area under the ROC curve (AUC) was 0.938 (P < 0.001). ROC: receiver operating characteristic

The Pearson correlation coefficient between ACT and SAQ-1 scores was 0.813 (P < 0.001), Kendall’s Tau-b correlation coefficient was 0.684 (P < 0.001), and the Spearman correlation coefficient was 0.828 (P < 0.001) (Table 3). These correlations indicate a strong positive correlation between ACT and SAQ-1 scores. The cross-tabulation analysis of the ACT and SAQ-1 scores (Table 4) showed that asthma patients with higher ACT score > 19 (indicating well-controlled asthma) had higher SAQ-1 scores 7-10, showing a strong correlation between these two scores. In contrast, patients with ACT scores ≤18 (indicating not well-controlled asthma) had a wider range of SAQ-1 scores (4-10), suggesting a weaker correlation between the two scores within these ranges. The ROC curve showed a strong area under the ROC curve (AUC) for SAQ-1 was 0.938 (standard error = 0.022, P < 0.001), with a 95% confidence interval of 0.895 to 0.982 (Figure 3). An SAQ-1 score ≤7 has a sensitivity (85.0%) and specificity (91.4%), correlating with an ACT score ≤ 19 (indicating not well-controlled asthma) (Table 5).

**Table 3:** Correlation coefficients (r) were used for analysis including Pearson, Kendall’s Tau-b, and Spearman with a range from -1 to 1. Positive values close to 1 show a strong positive relationship, values near 0 indicate little or no relationship, and negative values close to -1 show a strong negative correlation. The area under the ROC curve (AUC) was used to determine the diagnostic accuracy of SAQ-1, with the corresponding p-value. Sensitivity and specificity for SAQ-1 cutoff points are noted and do not have associated p-values, as no statistical test was used.

Statistical Test	Result	Test Statistic	p-value
Pearson correlation coefficient (r)	Strong positive correlation	r = 0.813	p < 0.001
Kendall’s Tau-b correlation coefficient (r)	Moderate positive correlation	r = 0.684	p < 0.001
Spearman correlation coefficient (r)	Strong positive correlation	r = 0.828	p < 0.001
Area under the ROC curve (AUC) value	Excellent diagnostic accuracy	AUC = 0.938	p < 0.001
95% confidence interval for AUC value	0.895-0.982	N/A	N/A
Sensitivity/specificity for SAQ1 ≤ 7	Sensitivity = 85% Specificity = 91.4%	N/A	N/A
Sensitivity/specificity for SAQ-1 ≤ 6	Sensitivity = 67.5% Specificity = 94.2%	N/A	N/A

**Table 4:** Cross-tabulation analysis of ACT scores (≤18, 19, and 20–25) vs. SAQ-1 scores (0–3, 4–6, and 7–10); (n = 115 patients).

ACT score	SAQ-1 score	Frequency n (%) within ACT score
≤18	0–3	11 (14.5%)
	4–6	42 (55.2%)
	7–10	23 (30.3%)
19	0–3	0 (0.0%)
	4–6	1 (25.0%)
	7–10	3 (75.0%)
20–25	0–3	0 (0.0%)
	4–6	3 (8.6%)
	7–10	32 (91.4%)

**Table 5:** Sensitivity, positive predictive value, specificity, and negative predictive value for SAQ-1 scores in relation to ACT scores ≤ 19 based on ROC curve analysis. SAQ-1 score ≤ 7 had the best sensitivity (85.0%) and specificity (91.4%) indicating not well-controlled asthma. SAQ-1: Subjective Asthma Questionnaire-1; ACT: Asthma Control Test

Metric	SAQ-1 ≤7	SAQ-1 ≤ 6
Sensitivity	85.0%	67.5%
Positive predictive value	95.7%	96.4%
Specificity	91.4%	96.4%
Negative predictive value	72.7%	55.9 %

 Secondary analysis

**Figure 4:**
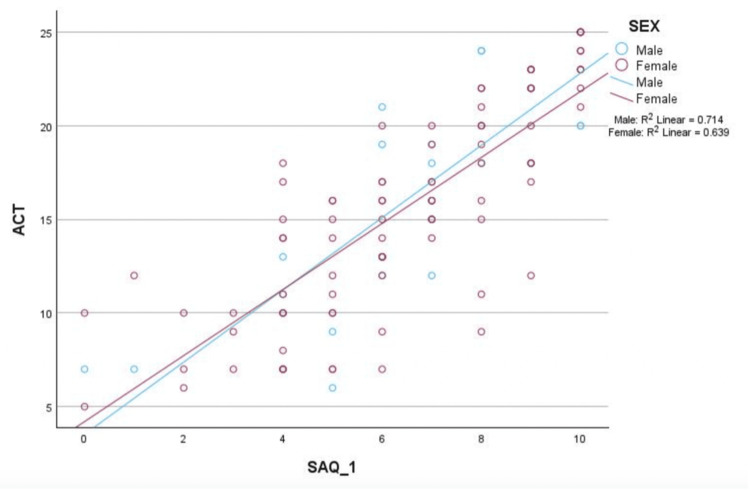
Scatter plot of ACT vs SAQ-1 scores by gender. The R2 = coefficient of determination (range 0-1) analyses showed that gender did not affect the correlation between ACT and SAQ-1 scores and that SAQ-1 scores are a good predictor of ACT scores in both males and females. Male patients: R2 linear = 0.714; Female patients: R2 linear = 0.639.

We used a scatter plot and coefficient of determination (R², ranging from 0 to 1) to compare the relationship between the SAQ-1 and ACT scores by gender (Figure 4). The coefficient of determination (R²) for males was 0.714 and for females was 0.639. Pearson correlation coefficients were used to determine the relationships (+1 to -1, positive or negative correlations) between the demographic and clinical variables and ACT and SAQ-1 scores (Table 6). Asthma severity was significantly and strongly negatively correlated with both ACT (r = −0.747, P < 0.001) and SAQ-1 (r = −0.655, P < 0.001), indicating that more severe asthma was associated with lower ACT and SAQ-1 scores. Race was significantly and weakly negatively correlated with ACT (r = −0.227, P = 0.015) and SAQ-1 (r = −0.199, P = 0.034), with Black patients having lower scores compared to White patients. Other demographic and clinical characteristics, including age, gender, BMI, smoking status, pulmonary function tests, and elevated serum eosinophils, did not show any statistically significant correlations with ACT or SAQ-1 scores (Table 6).

**Table 6:** Secondary analysis used Pearson correlation coefficients (ranging from −1 to +1, indicating negative or positive correlations) to assess relationships between ACT and SAQ-1 scores and various demographic and clinical characteristics. Demographics included age, gender, and race, while clinical characteristics included asthma severity, elevated eosinophils, BMI, weight, smoking status, and pulmonary function tests (FEV1, FVC, TLC, RV, and DLCO corrected). Asthma severity and race were significantly negatively correlated with both ACT and SAQ-1 scores, while other demographic and clinical characteristics were not found to be significant. n = 115 patients. FEV1: forced expiratory volume in 1 second; FVC: forced vital capacity; TLC: total lung capacity; RV: residual volume; DLCO: diffusion capacity of the lungs for carbon monoxide

Variable	Associations with ACT (Pearson correlation, P-value)	Associations with SAQ-1 (Pearson correlation, P-value)	Associations with ACT and SAQ-1
Age	0.030, 0.754	0.052, 0.581	Not significant
Asthma severity	−0.747, <0.001	−0.655, <0.001	Strongly negative
BMI	−0.127, 0.176	−0.114, 0.224	Not significant
Gender (Male/female patients)	−0.115, 0.218	−0.103, 0.274	Not significant
Elevated serum eosinophils	−0.040, 0.729	−0.044, 0.703	Not significant
Race (Black/White patients)	−0.227, 0.015	−0.199, 0.034	Weakly negative
FEV-1	0.073, 0.525	0.016, 0.888	Not significant
FVC	0.054, 0.636	−0.058, 0.609	Not significant
TLC	0.085, 0.464	0.057, 0.624	Not significant
RV	0.068, 0.564	0.054, 0.650	Not significant
DLCO corrected	0.136, 0.250	0.186, 0.113	Not significant
Smoking	−0.050, 0.613	−0.058, 0.560	Not significant
Weight	−0.105, 0.262	−0.025, 0.793	Not significant

## Discussion

The GINA and NAEPP guidelines emphasize the importance of regularly assessing asthma control at each patient visit to guide management decisions [2,3,13]. Regular assessment is crucial for optimizing treatment plans and preventing asthma exacerbations [2,3]. Common asthma screening tools include the ACT and ACQ, both of which have been validated in numerous studies [7-14]. The ACT is the most widely used questionnaire in the US, while the ACQ is more commonly applied in the United Kingdom [5,6]. However, non-pulmonologists may find these tools challenging to interpret, suggesting that a simpler tool like the SAQ-1 could be more practical for routine use [8].

In this study, we report the first use of the SAQ-1 asthma scoring system and compare its performance with the standard ACT score. In our patient cohort, the mean ACT score was 15.79 (standard deviation, 5.65), indicating not well-controlled asthma, while the mean SAQ-1 score was 6.50 (standard deviation, 2.53). Correlation analyses, including Pearson, Kendall’s Tau-b, and Spearman methods, demonstrated strong positive correlations between the ACT and SAQ-1 scores (P < 0.001), indicating that higher ACT scores (reflecting better asthma control) were associated with higher SAQ-1 scores.

The ROC curve analysis further supports the use of the SAQ-1 score in asthma assessments, with an AUC of 0.938, indicating excellent diagnostic accuracy. At a threshold of ≤7, the SAQ-1 demonstrated a sensitivity of 85.0% and specificity of 91.4%, aligning with the ACT score cutoff of ≤19 for identifying patients with not well-controlled asthma. Similar findings have been reported in studies validating other asthma assessment tools [14], which underscores the potential of the SAQ-1 as a practical screening tool.

Regarding demographic and clinical characteristics, our analysis found no significant associations between ACT or SAQ-1 scores and variables such as age, gender, weight, or BMI (Table 6). A significant negative correlation was observed between asthma severity and both ACT and SAQ-1 scores, indicating that as asthma severity increased, the control scores decreased. This result is consistent with other studies showing that increased asthma severity is associated with lower control scores [10,11,14]. Additionally, a significant but weak negative association was found between race and asthma control scores, as Black patients had lower ACT and SAQ-1 scores than White patients. These disparities could reflect differences in healthcare access, environmental exposures, and socioeconomic factors that disproportionately affect Black populations [12].

A strength of this study is the use of multiple statistical analyses, which consistently showed strong correlations between SAQ-1 and ACT scores. This suggests that the SAQ-1 could be a practical tool for assessing asthma control in outpatient pulmonary settings.

Limitations

This study was conducted at a single academic pulmonary clinic with a limited sample size of 115 patients, predominantly from a Black population. These factors may limit the generalizability of the findings due to potential differences in clinical practice patterns across different settings. Additionally, while the SAQ-1 simplifies the assessment process with its single-item approach, it may not capture all aspects of asthma control, such as symptom frequency and the impact on daily activities, which are better addressed by multi-item tools like the ACT [8,14]. The reliance on patient self-reporting for both the ACT and SAQ-1 introduces the potential for recall bias, which could impact the accuracy of the results, as patients may not consistently remember and report symptoms accurately. Furthermore, the SAQ-1’s focus on patient perception may limit the depth of insights compared to the multi-question format of the ACT [8,14]. Future studies should involve larger, more diverse populations across multiple clinical settings to comprehensively validate the SAQ-1 score.

## Conclusions

In our study, the SAQ-1 score proved to be an effective tool for assessing asthma control, demonstrating a strong correlation with the well-validated ACT score. The SAQ-1 showed high sensitivity and specificity in identifying patients with not well-controlled asthma when compared to the ACT score. Additionally, correlations were observed between asthma severity and race for both scores, while no strong associations were found with other clinical or demographic factors. Future studies should evaluate the SAQ-1 across broader clinical settings, including both academic and private practices, to further validate its utility.
